# The effect of vibration training on delayed muscle soreness: A meta-analysis

**DOI:** 10.1097/MD.0000000000031259

**Published:** 2022-10-21

**Authors:** Yikun Yin, Jialin Wang, Kangqi Duan, Hejia Cai, Junzhi Sun

**Affiliations:** a College of Physical and Health Education, Guangxi Normal University, Guilin, China; b Institute of Sports Medicine and Health, Chengdu Sport University, Chengdu, China.

**Keywords:** delayed muscle soreness, meta-analysis, pressure pain threshold, serum creatine kinase, subjective pain, vibration training

## Abstract

Delayed onset muscle soreness (DOMS) is caused by unaccustomed exercise, especially eccentric exercise, and is highly likely to cause skeletal muscle injury. It mainly manifests as ultrastructural changes in skeletal muscle, as well as decreased muscle strength, muscle soreness, swelling, and elevated levels of creatine kinase (CK). Vibration training (VT) has been attracting increasing attention as a new type of rehabilitation therapy. It can effectively minimize the occurrence and relieve the symptoms of DOMS, reduce muscle stiffness and soreness, and reduce serum concentrations of CK and lactate dehydrogenase (LDH). This article systematically assessed the impact of VT on the mitigation of DOMS through a meta-analysis to provide updated evidence-based information.

**Methods:**

Electronic databases such as China Knowledge Network, VIP Electronics, PubMed, EBSCO, and Web of Science were searched to identify randomized controlled trials of VT on DOMS. Searches were performed from database creation to November 2021. The quality of the literature was assessed using the Cochrane Manual for the Systematic Review of Interventions, and meta-analyses were performed using RevMan 5.4 software.

**Results:**

VT intervention in DOMS was shown to effectively reduce subjective pain, improve pain tolerance, and accelerate the reduction of serum CK and LDH concentrations. Subgroup analysis of different test time periods showed that subjective pain decreased more significantly after 48 hours than after the other 2 time periods, and pain tolerance increased more significantly after 72 hours than the other 2 time periods; serum CK was significantly increased after 24 and 48 hours of intervention, but showed no significant change compared with the control group after 72 hours. Serum LDH decreased significantly after 24 hours of intervention, but there was no significant difference compared with the control group after 48 hours or 72 hours.

**Conclusion:**

VT effectively reduced the subjective pain sensation after DOMS, increased the pain threshold, reduced serum LDH and CK concentrations, and accelerated muscle damage repair compared with control interventions. However, the effect of improving the range of motion of the joints is not clear and should be studied further.

**Registration:**

number: INPLASY2021120115.

## 1. Introduction

Delayed onset muscle soreness (DOMS) occurs after high-intensity, high-load strength training or centrifugal contraction exercise, and is characterized by muscle soreness and stiffness; it can also impair muscle function, and decrease muscle strength and joint mobility.^[1,2]^ It usually peaks within 24 to 72 hours after activity, with symptoms gradually decreasing after 5 days and recovery occurring after around 1 week.^[3,4]^ DOMS may result from damage to the cytoskeleton, cell membranes, and microscopic muscle fibers in muscles after strenuous centrifugal exercise, leading to the production of large quantities of inflammatory mediators, and increased levels of serum creatine kinase (CK) and myoglobin.^[5,6]^ Previous studies showed the visual analog scale (VAS) and pressure pain threshold (PPT) to be the most common and reliable indicators of DOMS, in addition to blood and mechanical indicators such as serum CK and lactate dehydrogenase (LDH), surface electromyography,^[7]^ and isokinetic peak torque.^[8]^

Although recovery from DOMS can occur within 1 week, it greatly affects exercise participation, leading to a reduction in training effectiveness and exercise capacity; it may also increase the risk of injury during exercise, especially when returning to sport or training.^[9,10]^ Therefore, it is of great importance to select effective treatment modalities to gain relief and recovery from athletic muscle fatigue, promote improvement in physical exercise function, and reduce the risk of injury.

There are currently many tools for sports muscle recovery, including stretching, tui na, acupuncture, heat, foam axis rolling, intramuscular effect patches, and transcutaneous electrical stimulation,^[11–13]^ but their effectiveness is inconsistent. Although analgesics can be used, they have short-term effects and are at risk of abuse.^[14]^ Therefore, non-pharmacological adjunctive pain relief therapies have become increasingly popular in recent years.

Vibration training (VT) is gradually gaining attention as a new type of rehabilitation therapy.^[15]^ VT acts on the body through mechanical vibrations of different amplitudes and frequencies generated by a platform to link the skeletal–muscular–neural chain.^[16,17]^ It stimulates neuromuscular excitability, increases the frequency of evoked muscle action potentials, enhances neuromuscular control, and allows higher muscle grooming and the recruitment of muscle fibers, thereby reducing muscle stiffness and soreness, and decreasing the occurrence of delayed muscle soreness.^[18]^

To date, 3 systematic reviews^[19–21]^ have reported the effect of VT on DOMS; however, these have several shortcomings, including an incomplete literature search and single outcome indicators. Therefore, the present study reviewed the research literature on the effect of VT on DOMS remission through meta-analysis to provide updated evidence-based information.

## 2. Materials and methods

This systematic review protocol has been registered on INPLASY (registration number: INPLASY2021120115), and is available in full at https://inplasy.com/inplasy-2021-12-0115/). The protocol has been checked against the Preferred Reporting Items for Systematic review and Meta-Analysis Protocols checklist.^[22]^

### 2.1. Eligibility criteria

Studies were included if they were randomized controlled trials of the effect of VT on the treatment of DOMS. They were also required to include an experimental group treated with VT only (the vibration time, frequency, and duration were not limited), and a control group treated with stretching, massage, or no intervention. The participants were required to: be aged ≥ 18 years, in good physical condition, and with no contraindications to exercise; have DOMS induced by exercise; and have no lower limb muscle pain, musculoskeletal disorders, neurological or cardiovascular diseases. Studies were excluded if they: were written in languages other than English or Chinese; involved experimental animals; were non-clinical trials or experiments of non-interventional design; and were studies for which data could not be extracted effectively and the original text was not available.

Primary evaluation indicators were VAS, PPT, and serum CK; secondary evaluation indicators were LDH and knee mobility (ROM).

### 2.2. Electronic data sources

The following electronic databases were searched from the time of their creation to November 2021: China Knowledge Network, Weipu Electronics, PubMed, Cochrane Library, EBSCO, and the Web of Science.

### 2.3. Search strategy

The search was performed in PubMed using the following terms: whole-body vibration training, vibration training, vibration, VT, WBVT, delayed onset muscle soreness, muscle soreness, muscle damage, and DOMS. The search strategy is shown in Table 1. Different databases have different characteristics and different retrieval strategies.

**Table 1:** Search strategy for the PubMed database.

Appendix I Search strategy used in Pubmed database	
Number	Search terms
#1	whole-body vibration training [MeSH Major Topic]
#2	vibration training [MeSH Major Topic]
#3	vibration [MeSH Major Topic]
#4	VT[MeSH Major Topic]
#5	WBVT [MeSH Major Topic]
#6	or/#1-#5
#7	delayed onset muscle soreness
#8	muscle soreness,
#9	muscle damage,
#10	DOMS
#11	or/#7-#10
#12	#6 AND #11

### 2.4. Literature screening and information extraction

EndNote X9 software was used to cull, merge, and screen the retrieved literature. This was performed by 2 researchers, and the following information was extracted after reading the abstract and full text: first author, year of publication, sample size, intervention, vibration protocol, test assessment index, and test duration. If the literature lacked any of this information, it was obtained by emailing the authors; studies lacking information from other sources or whose authors did not respond were excluded.^[23]^ If the 2 researchers disagreed or disputed the same piece of literature, a third researcher arbitrated the decision.

### 2.5. Assessment of risk of bias for study quality assessment

The risk of bias was assessed by 2 researchers for methodological quality following the Risk of bias assessment tool in the Cochrane Handbook for Systematic Evaluation of Interventions^[24]^ for the screened literature in 6 areas: random allocation sequence, allocation scheme concealment, blinding, incomplete outcome data, selective reporting of results, and other issues. The quality assessment was performed independently; in the case of disagreement, a third investigator’s opinion was consulted.

### 2.6. Statistical analysis

Data on the assessment indicators of the included literature were processed using meta-analysis software RevMan v.5.4 provided by the Cochrane Collaboration Network. The mean difference (MD) or standardized mean difference (SMD) and 95% confidence interval (CI) were used for the analysis of the outcomes of all included literature involving continuous variables. Heterogeneity between the outcomes of included studies was analyzed using the χ^2^ test (test level α = 0.1), while the magnitude of heterogeneity was determined quantitatively by junction *I*^2^. Meta-analysis was performed using a fixed effects model if there was no heterogeneity in the studies. If statistical heterogeneity between studies was identified, the sources were further analyzed, and meta-analysis was performed using a random effects model after excluding the effect of significant clinical heterogeneity. The level of meta-analysis was set at α = 0.05. Funnel plots were used for the included studies to assess the role of publication bias as the number of studies exceeded 10.^[25]^

### 2.7. Ethics and dissemination

This study used published data that were not linked to individuals, so does not require ethical approval.

## 3. Results

### 3.1. Search results and literature screening

**Figure 1.**
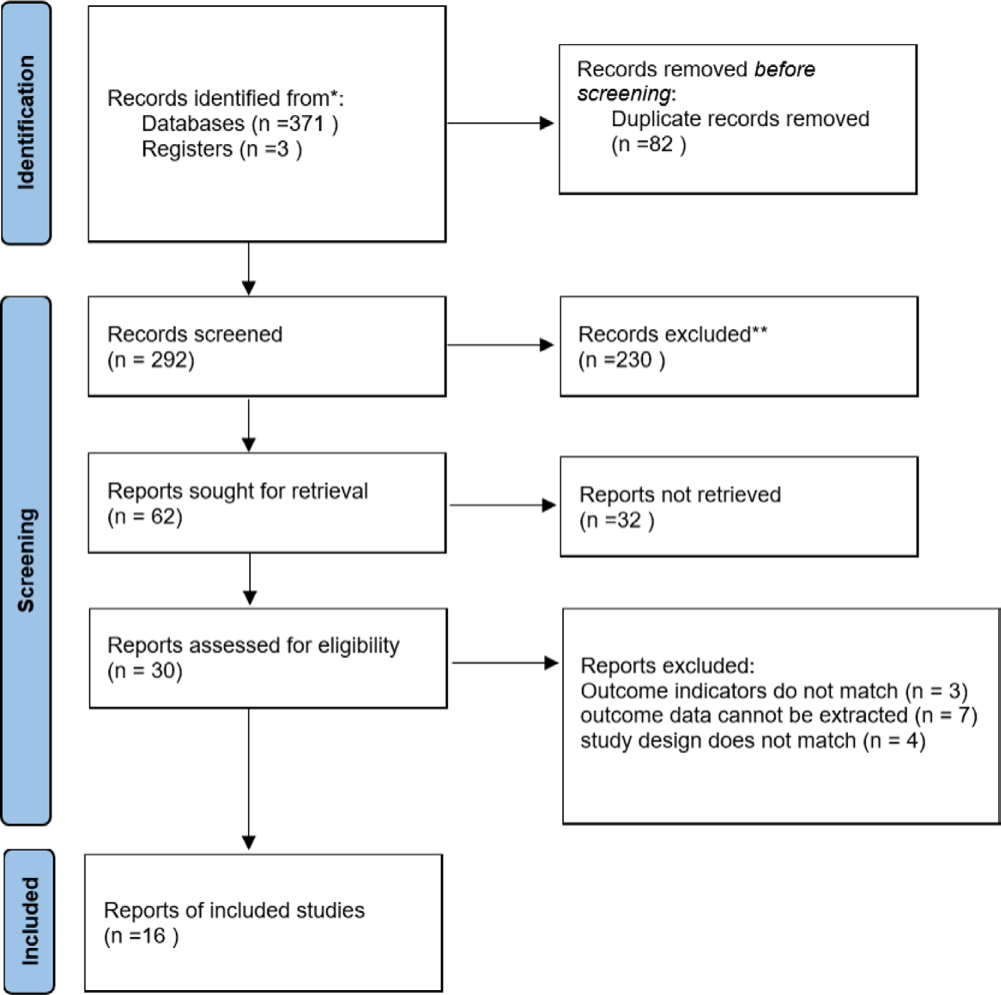
Literature screening process. *PubMed (194), CNKI (7), Web of science (159), EBSCO (5), and VIP (6).

Database searches initially retrieved 371 articles, and a further 3 articles were obtained from other sources. The retrieved articles were subsequently merged and 82 duplicates were excluded using EndNote X9. A total of 62 articles were screened by reading the title and abstract, and 30 by reading the full text. Sixteen articles were finally included in the meta-analysis based on inclusion criteria. The literature screening process and results are shown in Figure 1 and Table 2.^[26–41]^

**Table 2:** Information extraction from articles.

Author	Country	DOMS modeling method	Modeling part	Participants		Year		Vibration intervention	Control measures	Outcomes	Data time point (h)
				T	C	T	C				
Shen YH 2017^[23]^	China	Frog-leaping exercise (15 per group/group, 8 groups). Rest time 3 min	Knee extensor muscles	6	6	20.5 (1.5)	20.5 (1.5)	60 s, frequency 35 Hz, amplitude 2 mm, 6 min in total	No intervention	①	24, 48, 72
Fan XJ 2013^[24]^	China	Muscle isokinetic eccentric resistance training, 10 reps/group, 6 groups, 3 min rest time between groups	Knee extensor muscles	15	15	23.52 (2.35)	22.64 (7.37)	Vibration in semi-squatting position for 1 min	–	①②③	24, 48, 72
Song FM 2017^[22]^	China	Treadmill-run downhill for 30 min at −10°	Knee extensor muscles	9	9	–	–	Frequency 30 Hz, amplitude 1.5 mm, 9 min	–	①②③ ④⑤	24, 48, 72
Zhong GY 2019^[21]^	China	Full squat frog jump 15 times/group + weight-bearing half squat jump 30 times/group, 10 groups, rest 120 s between groups	Knee extensor muscles	19	18	19.3 (1.0)	19.0 (1.2)	Frequency 50 Hz, amplitude 3 mm, 10 min	No intervention	①	24, 48, 72
Magoffin R D 2020^[25]^	America	Maximum strength eccentric contraction, 10 times/group, 30 groups	Knee extensor muscles	15	15	22.7 (2.9)		Frequency 40 Hz, amplitude 2.05 mm, 5 min	No intervention	⑤	24, 48, 72
Dabbs N C 2015^[26]^	America	Squat with 40% of your body weight, 4 groups, rest for 1 min between groups	Knee extensor muscles	16	14	21.0 (1.9)	22.00 (1.97)	Frequency 30 Hz, amplitude 2–4 mm	No intervention	①②⑤	24, 48, 72
Aminian-Far A 2011^[27]^	Iran	Maximum strength eccentric contraction, 10 times/group, 6 groups, rest 3 min between groups	Knee extensor muscles	15	17	21.46 (2.66)	21.88 (1.93)	Frequency 35 Hz, amplitude 5 mm, 5 min	No intervention	①②⑤	24, 48, 72
Fuller J 2015^[29]^	Australia	Muscle isokinetic eccentric resistance training 100 times	Knee extensor muscles	25	25	22.5 (3.7)		Frequency 3 Hz, 20 min	Massage stretch	①③	24, 48, 72
Timon R 2016^[31]^	Leah	5-minute warm-up (30% 1 RM) and 4 sets of 5 repetitions of 120% 1RM maximum intensity eccentric contraction, 4 min rest between sets	Quadriceps	10	10	24.2 (0.5)	23.4 (1.4)	Frequency 12 Hz, amplitude 4 mm, 1 min/group, 3 groups, 30 s recovery between groups	No intervention	①③	24, 48
Rhea M R 2009^[32]^	Spain	Self-weight squat, lower extremity resistance and eccentric exercise, 10 sprint runs with an interval of 60 s	Lower limb muscles	8	8	36.6 (2.1)		For 30 s, the frequency is 50 Hz; the amplitude is 2 mm, and for 60 s, the frequency is 35 Hz; the amplitude is 2 mm	Stretch	①	24, 48, 72
Bakhtiary A H 2007^[33]^	Spain	Treadmill-run downhill for 30 min at −10°, 4 km/h	Lower limb muscles	25	25	20.6 (1.9)	20.6 (2.1)	Frequency 50 Hz,1 min	No intervention	①②③	24
Harold Akehurst 2021^[34]^	Iran	60% 1RM weight for knee extension exercises, 10 reps/group, 4 groups	Quadriceps	8	8	26	27	Frequency 30 Hz, amplitude 4 mm, 2 min	Stretch	①	24, 48, 72
Kim YS 2007^[36]^	Germany	Biceps use a weight equivalent to 70% of the maximum number of repetitions, slowly descend at the same speed and lift with assistance, 10 reps/group, 7 groups, rest 4 min between groups	Bicipital muscle of arm	7	7	18–25		Frequency 26 Hz, amplitude 3 mm, 11 min	Ultrasound	①②	24, 48, 72
Cochrane D J 2017^[28]^	South Korea	Muscle isokinetic eccentric resistance training, 6 times/group, 10 groups, rest 2 min between groups	Elbow flexion muscle group	13	13	21.7 (2.6)		Frequency 120 Hz, 15 min	No intervention	①② ③⑤	24, 48, 72
Kim J Y 2017^[30]^	New Zealand	Muscle eccentric contraction training, 15 times/group, 5 groups, 1 min rest between groups	Elbow flexion muscle group	10	10	20		Frequency 60 Hz, 5 min	No intervention	②③④	24, 48, 72
Cecilia Drennen 2014^[35]^	South Korea	Squat, 4 sets, 1 min rest between sets	Quadriceps	15	15	21 (1.9)	21 (1.9)	No intervention	No intervention	②	24, 48, 72

①Pain visual analogue score (VAS), ②Pressure pain threshold (PPT), ③Sera creatine kinase (CK), ④Lactate dehydrogenase (LDH), ⑤Knee range of motion (ROM).

T = experimental group, C = control group, - = means not mentioned.

### 3.2. Basic characteristics of the included studies

The 16 articles included 431 subjects. Most of the included studies (75%) were published between 2012 and 2021.

### 3.3. Methodological quality evaluation of the included literature

**Figure 2.**
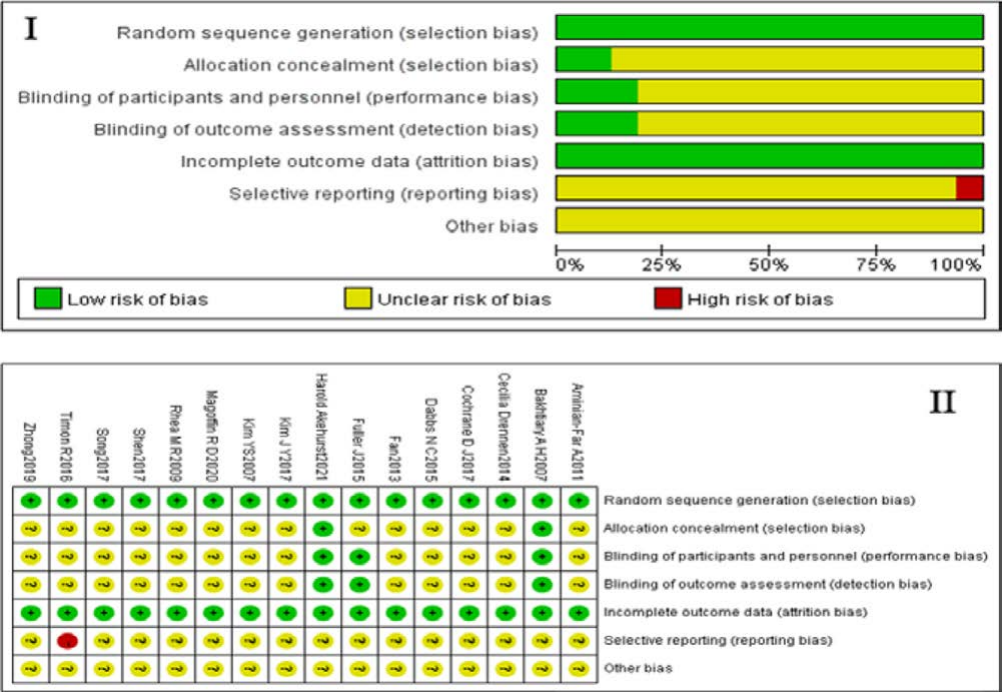
The results of the risk of bias evaluation of the included studies. Figures I and II are respectively the overall risk of bias in the included studies and the specific risk assessment of bias in each study. Green means low risk of bias, red means high risk of bias, and yellow means ominous risk of bias. “+” means low risk of bias, “-” means high risk of bias, and “?” means unknown risk of bias.

Risk assessment findings of the 16 randomized controlled trials are shown in Figure 2.

### 3.4. Meta-analysis

#### 3.4.1. VAS for pain

**Figure 3.**
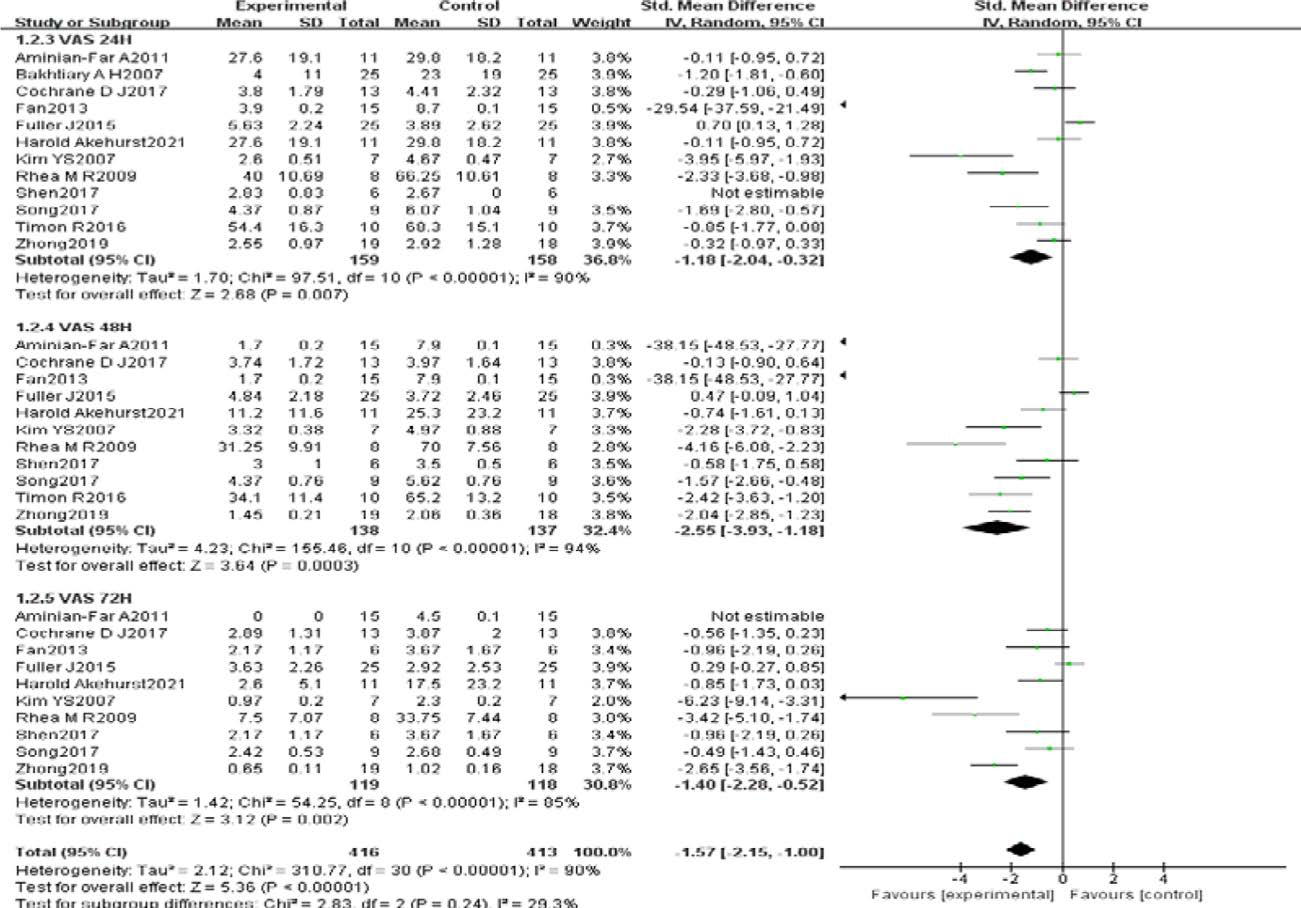
Meta-analysis of the impact of subjective pain after VT intervention in DOMS. DOMS = delayed onset muscle soreness, VT = vibration training.

Twelve of the included studies^[25–29,32–38,40,41]^ evaluated the effect of VT on subjective pain in DOMS using the pain VAS score in a total of 317 subjects. Statistical heterogeneity was detected (χ^2^ = 310.77, *P* < .01, *I*^2^ = 90%), so the random effects model was chosen. Meta-analysis results showed that SMD = –1.57, 95% CI (–2.15, –1.00), *P* < .01, indicating that VT was significantly better than the control intervention in relieving DOMS pain. The SMD was combined because of the use of different units of measurement among studies. Subgroup analysis of different testing times revealed that VT was significantly better than the control intervention at relieving pain at 24 hours (SMD = –1.18, 95% CI [–2.04, –0.32], *P* < .01), 48 hours (SMD = –2.55, 95% CI [–3.93, –1.18], *P* < .01), and 72 hours (SMD = –1.40, 95% CI [–2.28, –0.52], *P* < .01) (Fig. 3).

#### 3.4.2. PPT

**Figure 4.**
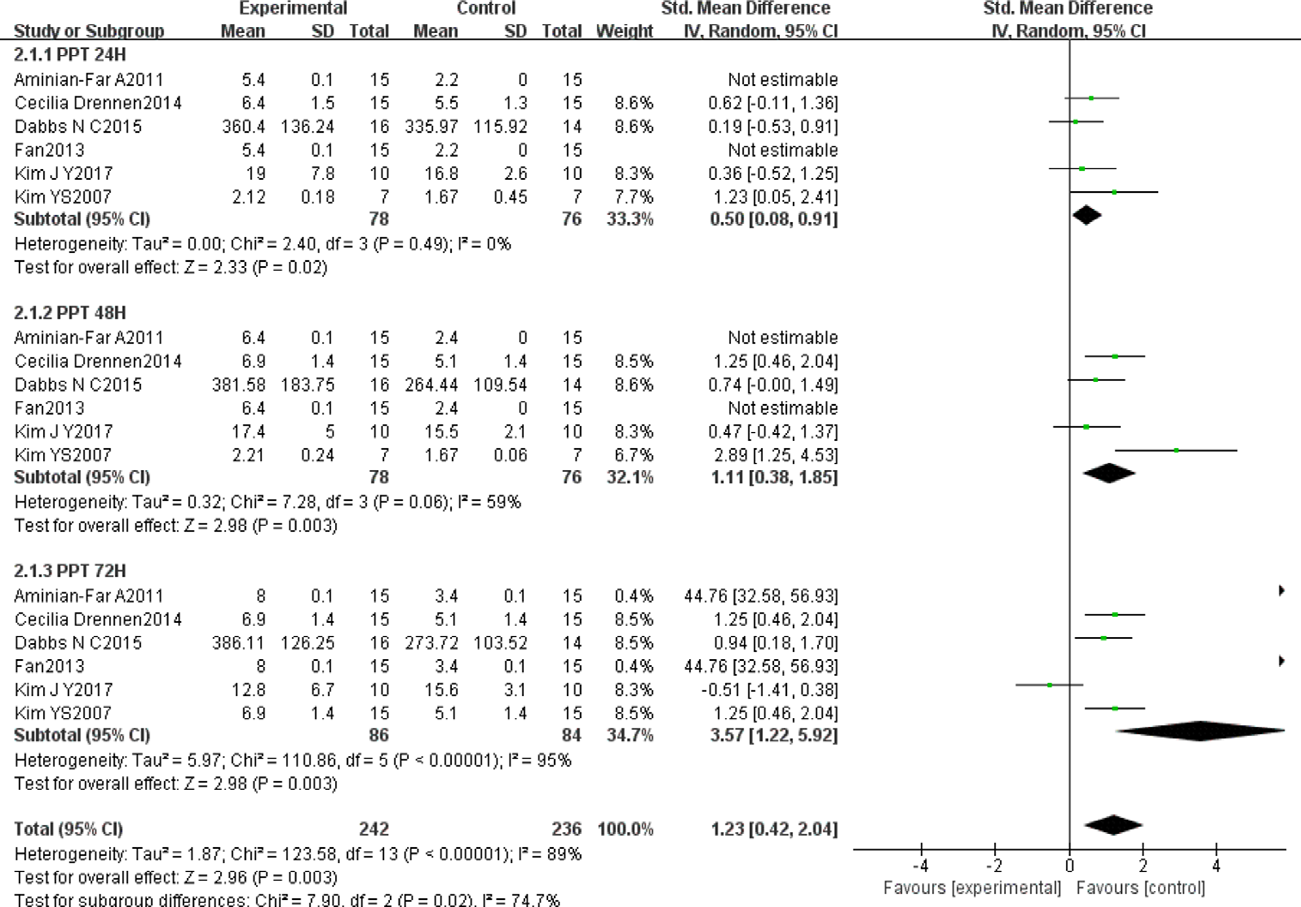
Meta-analysis of the effect of VT on the tenderness threshold after DOMS intervention. DOMS = delayed onset muscle soreness, VT = vibration training.

Six of the included studies^[29,31,32,35,40,41]^ evaluated the effect of VT on the degree of pain tolerance in DOMS using PPT in a total of 154 subjects. Statistical heterogeneity was again detected (χ^2^ = 123.58, *P* < .01, *I*^2^ = 95%), so the random effects model was chosen. Meta-analysis showed that SMD = 1.23, 95% CI (0.42, 2.04), *P* < .01, indicating that VT was significantly better than the control in improving PPT after DOMS. As before, the SMD was combined because different units of measurement were used. Subgroup analysis of different testing times revealed that VT was significantly better than the control at 24 hours (SMD = 0.50, 95% CI [0.08, 0.91], *P* = .02), 48 hours (SMD = 1.11, 95% CI [0.3, 1.85], *P* < .01), and 72 hours (SMD = 3.57, 95% CI [1.22, 5.92], *P* < .01) (Fig. 4).

#### 3.4.3. Serum CK

**Figure 5.**
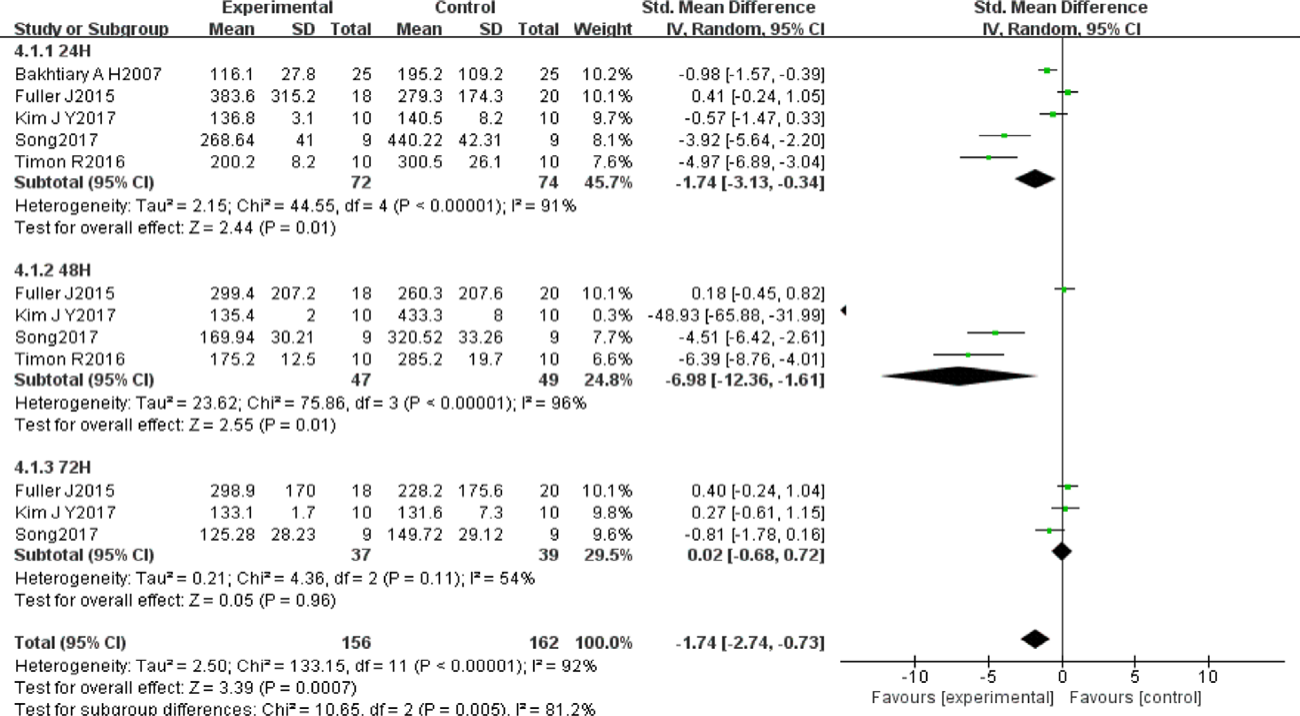
Meta-analysis of the influence of CK concentration after VT interferes with DOMS. CK = creatine kinase, DOMS = delayed onset muscle soreness, VT = vibration training.

Five of the included studies^[27,34–36,38]^ evaluated changes in CK concentrations after VT intervention for DOMS in a total of 146 subjects. Statistical heterogeneity was detected (χ^2^ = 133.15, *P* < .01, *I*^2^ = 92%), so the random effects model was chosen. Meta-analysis showed that SMD = –1.74, 95% CI (–2.74, –0.73), *P* < .01, indicating that the reduction in CK after VT intervention for DOMS was significantly better than in the control group. SMD was combined as different units of measurement had been used. Subgroup analysis of different testing times revealed that VT was superior to controls in reducing CK at 24 hours (SMD = –1.74, 95% CI [–3.13, –0.34], *P* = .01) and 48 hours (SMD = –6.98, 95% CI [–12.36, –1.61], *P* = .01) post-exercise, but there was no significance at 72 hours (SMD = 0.02, 95% CI [–0.68, 0.72], *P* = .96) (Fig. 5).

#### 3.4.4. LDH

**Figure 6.**
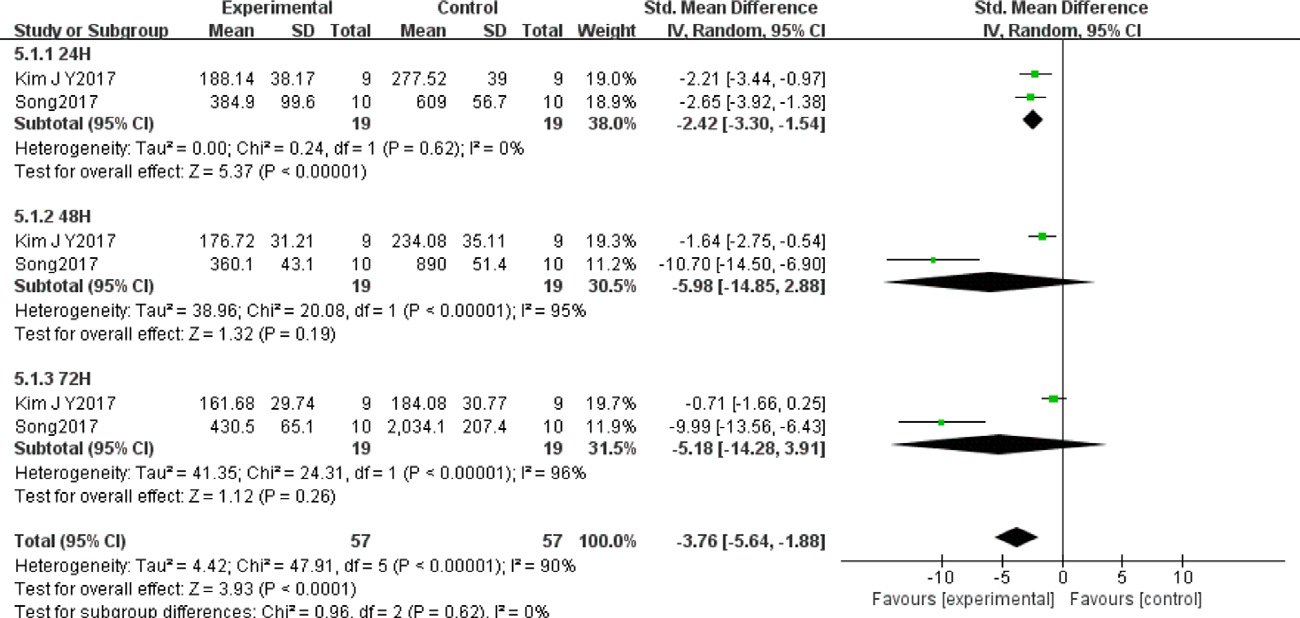
Meta-analysis of the influence of LDH concentration after VT interferes with DOMS. LDH = lactate dehydrogenase, DOMS = delayed onset muscle soreness, VT = vibration training.

Two of the included papers^[27,35]^ evaluated changes in LDL concentrations after VT intervention for DOMS in 38 subjects. Statistical heterogeneity was confirmed (χ^2^ = 47.91, *P* < .01, *I*^2^ = 90%), so random effects model analysis was chosen. Meta-analysis showed that SMD = –3.76, 95% CI (–5.64, –1.88), *P* < .01, indicating that the reduction in LDH after VT intervention for DOMS was significantly better than in the control group. SMD was combined because different units of measurement were employed. Subgroup analysis of different testing times revealed that VT was superior to the control in reducing LDH 24 hours post-exercise (SMD = –2.42, 95% CI [–3.30, –1.54], *P* < .01), but there was no significant difference after 48 hours (SMD = –5.18, 95% CI [–14.85, 2.88], *P* = .19) or 72 hours (SMD = –5.18, 95% CI [–14.28, 3.91], *P* = .26) (Fig. 6).

#### 3.4.5. Knee ROM

**Figure 7.**
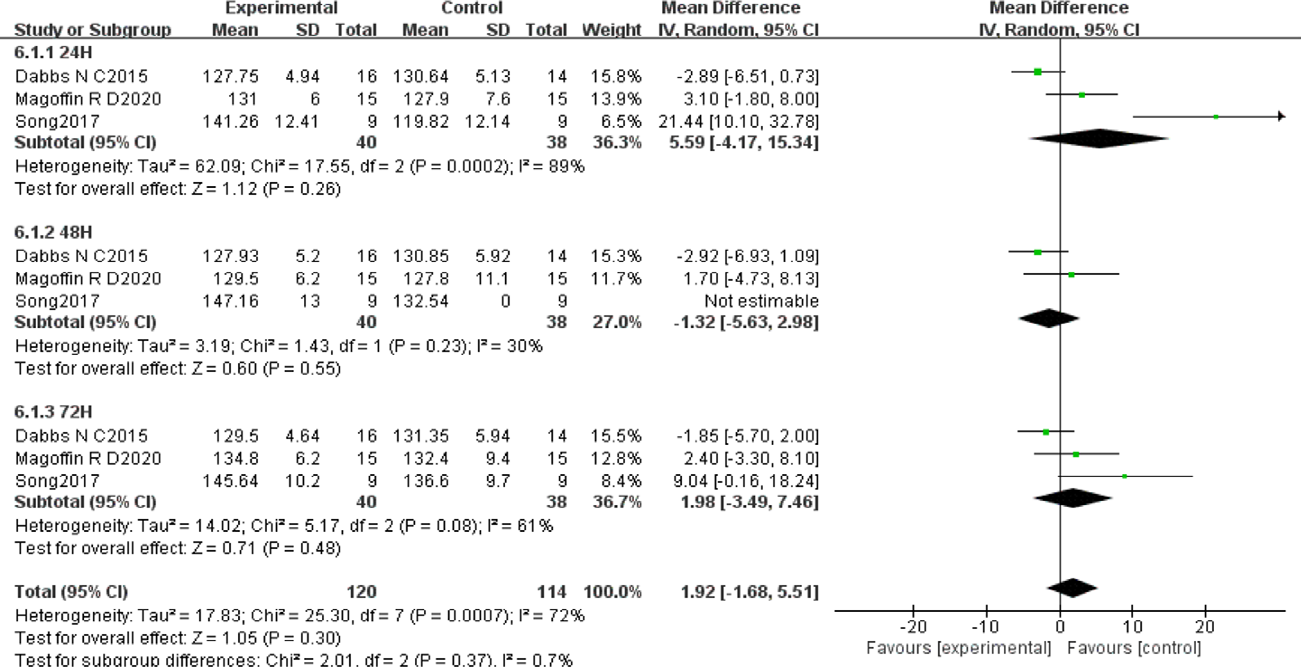
Meta-analysis of the influence of VT intervention on DOMS knee range of motion. DOMS = delayed onset muscle soreness, VT = vibration training.

Three papers^[27,30,31]^ reported changes in knee mobility after VT on DOMS intervention in 78 subjects. Statistical heterogeneity was detected (χ^2^ = 25.30, *P* ≤ .01, *I*^2^ = 72%), so a random effects model was chosen for analysis. Meta-analysis showed that MD = 1.92, 95% CI (–1.68, 5.51), *P* = .30, indicating that VT did not outperform the control in improving knee mobility. Subgroup analysis of different testing times confirmed that VT was not significantly better than control interventions at improving knee mobility after 24 hours (MD = 5.59, 95% CI [–4.17, 15.34], *P* = .26), 48 hours (MD = –1.32, 95% CI [–5.63, 2.98], *P* = .55), or 72 hours (MD = 1.98, 95% CI [–3.49, 7.46], *P* = .48) (Fig. 7).

### 3.5. Publication bias

**Figure 8.**
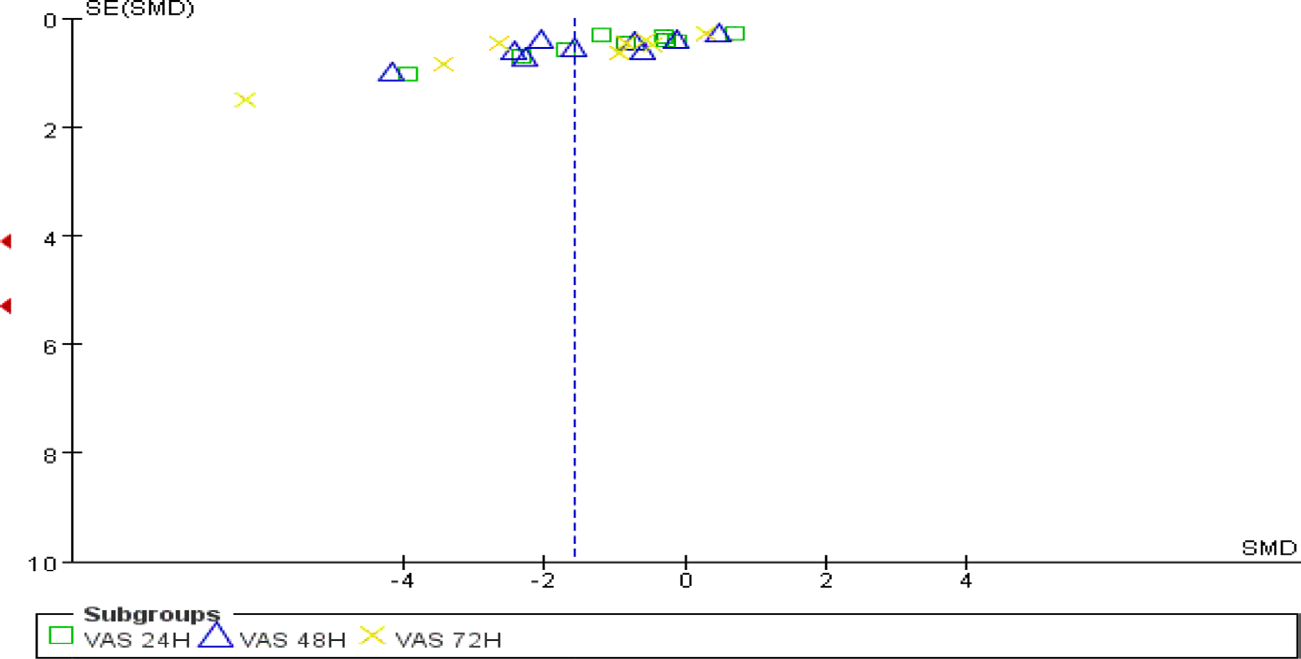
The risk of publication bias in the study of VT’s mitigation effect on DOMS. DOMS = delayed onset muscle soreness, VT = vibration training.

Funnel plot analysis of the included studies using the VAS score for pain as an indicator showed that the plots were largely symmetrical, indicating there was no significant publication bias and that the meta-analysis results are reliable (Fig. 8).

## 4. Discussion

DOMS is caused by unaccustomed exercise, mainly after high-intensity centrifugal exercise, and results in skeletal muscle injury and ultrastructural changes which manifest as muscle soreness, stiffness, swelling, decreased strength, and elevated serum CK concentrations.^[3,19,42]^ Of the many published DOMS mechanisms, the most generally accepted favors the mechanical injury theory, the inflammation theory, and other factors acting together to cause DOMS.^[1,43–45]^ Following high-intensity centrifugal exercise, muscle contractile structures within skeletal muscle are at their least stable, and myofilaments within muscle fibers fail to coordinate movement effectively. This results in excessive stress on single or weaker muscle fibers, overstretching to the point of tearing, and damage to the muscle membrane, leading to increased intracellular Ca^2+^ concentrations, calcium overload phenomena activating calpain, the destruction of protein structure, protein degradation, autophagy, and inflammatory responses.^[46,47]^

A total of 5 outcome indicators were included in the present study; primary evaluation indicators were the VAS for pain, PPT, and serum CK, while secondary evaluation indicators were LDH concentrations and knee ROM. Three published systematic reviews^[19–21]^ were analyzed, as shown in Table 3. To address previous shortcomings,^[38,49,50]^ we include 16 studies written in Chinese or English literature for analysis. Our findings revealed that after performing VT intervention in DOMS, VAS and CK and LDH concentrations decreased significantly relative to controls, and PPT increased significantly, but there was no significant improvement in knee mobility.

**Table 3:** Analysis of 3 published systematic reviews.

Author/publication year	Type and quantity of included documents	Outcomes	Conclusion	Limitation
Niu XR 2020^[49]^	9 randomized controlled trials	VAS, PPT, CK, ROM, PT	Vibration can relieve the subjective pain after DOMS, increase the pain threshold, promote muscle recovery, and improve muscle micro-damage	(1) The included literature is only for modeling of lower limb muscles (2) Meta analysis results are highly heterogeneous (3) Unpublished bias analysis
Lu X 2019^[50]^	10 randomized controlled trials	VAS, CK	Vibration intervention can relieve DOMS and reduce serum CK concentration	(1) There are fewer outcome indicators (2) Unpublished bias analysis
Veqar Z 2014^[38]^	3 randomized controlled trials, 1 case report, 1 randomized crossover experiment, 3 experimental studies	VAS, PPT	Vibration therapy can increase proprioceptive neuromuscular function, increase muscle strength and potential hormonal response, thereby reducing pain, improving mood, and improving lymphatic circulation	(1) The number of randomized controls is small (2) Meta analysis has not been performed (3) All documents are in English

CK = creatine kinase, LDH = lactate dehydrogenase, PPT = pressure pain threshold, ROM = range of motion, VAS = visual analogue score.

The VAS is the most common index for evaluating subjective pain in DOMS subjects, and is often used in clinics. The PPT evaluates the ability to tolerate painful stimuli, with a higher pain threshold representing a greater ability to tolerate pain.^[7]^ In this study, our meta-analysis results showed that VT intervention for DOMS effectively reduced subjective pain, while subgroup analysis showed that subjective pain was reduced 24, 48, and 72 hours after the intervention; the extent of subjective pain reduction was greater after 48 hours than at the other 2 time periods.

The mechanism by which VT reduces subjective pain could involve vibration-induced increases in local blood and lymphatic circulation, which improve local muscle temperature and skin blood flow, thus reducing the release of pain-causing substances and promoting the rapid removal of metabolites such as lactic acid from the blood to reduce inflammation and relieve pain.^[37,47,48]^

In the 6 papers that reported the results of PPT assessment, pain tolerance was shown to increase significantly after VT intervention for DOMS compared with the control. Subgroup analysis revealed that pain tolerance increased at 24, 48, and 72 hours after the intervention, with a higher degree of increase after 72 hours. A proposed mechanism for increased pain tolerance might be that vibration stimulates skin receptors, which in turn activate inhibitory neurons in spinal nerves, decrease the speed of pain signal transmission, and reduce conduction to the brain. Vibration may also decrease creatine sensitivity and increase gamma neuron. According to the “gate control theory,” vibration improves sensory pathways of the nervous system, and “closes” the gate of pain information between the spine and the brain. This in turn reduces the input of nerve fibers that conduct nociception and relieve pain, increasing the amount of pain which reduces the input of nociceptive nerve fibers, relieves pain, and finally increases the pain tolerance.^[19,49]^

Serum CK and LDH are used as markers of muscle damage; they are significantly increased when skeletal muscle or cardiac myocytes are damaged, so are often used to evaluate muscle damage after centrifugal exercise.^[50–52]^ Our results show that VT intervention for DOMS reduced the CK concentration significantly faster than the control intervention, while subgroup analysis showed that levels were significantly decreased 24 and 48 hours after the intervention; however this difference was lost by 72 hours. Similarly, VT intervention for DOMS reduced LDH concentrations significantly faster than controls up to 24 hours after intervention.

Because the release of serum CK and LDH appears to be caused by cell membrane leakage after muscle fiber injury,^[1]^ and centrifugal exercise has the tendency to break muscle fibers and prevent the effective recruitment of motor units, it is possible that vibration induces the activation of potential motor units. This would enable sufficient motor unit recruitment,^[53]^ limiting muscle fiber breakage and accelerating recovery to a healthy state, thus repairing the ultrastructure of muscle fibers and reducing CK and LDH concentrations.^[54]^ High-frequency vibration has previously been shown to accelerate local muscle tissue blood circulation,^[55,56]^ increase CK levels and nutrient delivery, eliminate metabolic waste, and accelerate the repair and remodeling of damaged muscles.^[27]^ However, the effect of VT on the improvement of knee mobility was not significant in the present study, which was confirmed by subgroup analysis of different time periods.

Our meta-analysis has several limitations. First, only 16 studies were included, so the sample size was relatively small which could have introduced bias. Second, the level of heterogeneity between some of the studies was high, and this may have impacted on the reliability of our analysis. Finally, the included studies reported different modeling sites, methods of DOMS, and frequencies, amplitudes, and times of vibration stimulation. This could have reduced the argumentation strength and affected the study conclusion. Additionally, outcome evaluation indexes in different studies used various units of measurement, which may have interfered with the reliability of the outcome. Moreover, testing of the outcome indexes adopted 24, 48, and 72 hours timepoints, which cannot be comprehensively compared.

## 5. Conclusions

Our meta-analysis showed that VT effectively reduced subjective pain after delayed muscle soreness, improved the pain threshold, reduced serum LDH and CK concentrations, and accelerated muscle injury repair compared with control interventions. However, because of reported variations in vibration frequency, amplitude, and time settings for VT, and the high level of statistical heterogeneity among studies which resulted in a low level of evidence, our findings should be further corroborated. Future studies should increase the sample size, include more diverse test evaluation indexes, appropriately increase the test duration, conduct objective assessments to further corroborate the authenticity of the efficacy, and provide more evidence-based information for the clinical application of vibration training.

## Acknowledgments

We thank Sarah Williams, PhD, from Liwen Bianji (Edanz) (www.liwenbianji.cn/) for editing the English text of a draft of this manuscript.

## Author contributions

Conceptualization: Yinkun Yin.

Data curation: Jialin Wang, Hejia Cai.

Formal analysis: Yinkun Yin.

Methodology: Junzhi Sun.

Project administration: Yinkun Yin, Jialin Wang.

Resources: Kangqi Duan.

Software: Yinkun Yin, Hejia Cai.

Supervision: Junzhi Sun, Jialin Wang.

Visualization: Jialin Wang.

Writing – original draft: Yinkun Yin, Jialin Wang.

Writing – review & editing: Junzhi Sun.
